# Apolipoprotein E Genotype Linked to Spatial Gait Characteristics: Predictors of Cognitive Dual Task Gait Change

**DOI:** 10.1371/journal.pone.0156732

**Published:** 2016-08-03

**Authors:** Rebecca K. MacAulay, Ted Allaire, Robert Brouillette, Heather Foil, Annadora J. Bruce-Keller, Jeffrey N. Keller

**Affiliations:** 1 Department of Psychology, Louisiana State University, Baton Rouge, LA, United States of America; 2 Institute of Dementia Research and Prevention, Pennington Biomedical Research Center/LSU, Baton Rouge, LA, United States of America; University of Valencia, SPAIN

## Abstract

**Background:**

Developing measures to detect preclinical Alzheimer’s Disease is vital, as prodromal stage interventions may prove more efficacious in altering the disease’s trajectory. Gait changes may serve as a useful clinical heuristic that precedes cognitive decline. This study provides the first systematic investigation of gait characteristics relationship with relevant demographic, physical, genetic (Apolipoprotein E genotype), and health risk factors in non-demented older adults during a cognitive-load dual task walking condition.

**Methods:**

The GAITRite system provided objective measurement of gait characteristics in APOE-e4 “carriers” (n = 75) and “non-carriers” (*n* = 224). Analyses examined stride length and step time gait characteristics during simple and dual-task (spelling five-letter words backwards) conditions in relation to demographic, physical, genetic, and health risk factors.

**Results:**

Slower step time and shorter stride length associated with older age, greater health risk, and worse physical performance (*p*s < .05). Men and women differed in height, gait characteristics, health risk factors and global cognition (*p*s < .05). APOE-e4 associated with a higher likelihood of hypercholesterolemia and overall illness index scores (*p*s < .05). No genotype-sex interactions on gait were found. APOE-e4 was linked to shorter stride length and greater dual-task related disturbances in stride length.

**Conclusions:**

Stride length has been linked to heightened fall risk, attention decrements and structural brain changes in older adults. Our results indicate that stride length is a useful behavioral marker of cognitive change that is associated with genetic risk for AD. Sex disparities in motor decline may be a function of health risk factors.

## Introduction

Abnormal gait characteristics have been posited to be an early risk marker for dementia and Alzheimer’s Disease (AD) within older adults. Notably, the hazard ratio for a dementia disorder appears to be significantly higher in those who demonstrate declines in both cognitive and gait characteristics [1]. Research indicates that cognitive demands upon attention (as measured by dual task gait conditions) substantially impacts gait characteristics within both demented and relatively healthy older adults [1], [2], [3], [4], [5], [6]. An explanation for this relationship is that changes in gait and cognition share a common underlying neuropathophysiology [7]. Indeed, medical conditions, particularly vascular risk, associated with an increased risk of dementia have been linked to motor decline in older adults [8], [9]. Furthermore, the presence of multiple chronic medical conditions and greater overall disease burden as measured by physiological changes appears to contribute to gait changes in older adults [10].

In AD, vascular diseases as well as other processes that are independent of vascular risk appear to be involved in gait decline [11]. There is research that links the apolipoprotein E (APOE) e4 allele, the strongest known genetic risk factor for AD, to decreased lower extremity strength in older adults [12] and to an increased rate of decline in gait speed in older men but not women [13]. However, the underlying mechanisms involved in AD related gait changes remains uncertain. Therefore, identifying specific gait characteristics relationship with relevant genetic, demographic, and health risk factors in non-demented older adults serves to improve our understanding of pathological processes involved in gait and cognitive decline.

Gait is a dynamic process that involves cognitive control of motor processes, as evidenced by disruption of gait automaticity via dual tasks that divide attentional resources while walking. In this respect, examination of dual-task related gait changes might serve as a sensitive indicator of attention network functioning in older adults.

Differential associations between spatial and temporal gait characteristics with indicators of structural brain changes and the magnitude of associations with memory and executive functioning/attention have been found [3], [4], [5], [6], [14], [15], [16], [17], [18], [19], [20], [21], [22]. Although less studied than temporal aspects of gait, changes in spatial gait characteristics have been linked to a higher fall risk, gait decrements during dual tasks, and structural brain changes in older adults with and without cognitive impairments [11], [14], [15], [16], [17], [18], [19], [20], [21]. Specific gait characteristics (e.g., short stride length vs. slow swing time) demonstrate shared as well distinct risk factor profiles in older adults at risk of developing dementia [22]. There is also evidence that temporal and spatial gait aspects interact with one another, in which shortened stride length appears to give rise to slower walking times within both demented and healthy older adults [4], [20]. However, a comprehensive investigation into the interplay between dual-task related gait disturbances with relevant risk factors is lacking.

This study provides the first systematic investigation of temporal (step time) and spatial (stride length) gait characteristics during simple and dual task walking conditions in relationship to relevant demographic, physical, genetic, and health risk factors in non-demented older adults. Differential as well as shared patterns between stride length and step time gait characteristics with the relevant risk factors were expected. Specifically, advanced age and increased illness burden were expected to associate with slower step times and shorter stride lengths across conditions. It was also hypothesized that APOE–e4 carriers as compared to non-carriers would demonstrate shorter stride lengths and greater dual-task related changes in stride length even when the effects of other relevant risk factors were adjusted for in the analyses.

## Methods

### Participants

Participants are part of an on-going longitudinal study (Louisiana Aging Brain Study: LABrainS) that investigates the effects of aging upon cognitive processes and daily living functioning in relatively healthy older adults. Study design and procedures have been previously described [4]. Briefly LABrainS participants are recruited through regular outreach efforts throughout Louisiana. Eligibility criteria for this study requires that participants be willing to undergo annual cognitive assessment and are over the age of 60 with no existing diagnosis of dementia or cognitive impairment at the time of baseline screening. Exclusion criteria includes: a Geriatric Depression Scale score ≥ 6 (15 item version [23]), a history of neurological or untreated health conditions (e.g., cerebrovascular disease, Parkinson’s disease, and/or a traumatic brain injury, etc.) that might cause cognitive impairment. All participants have a Clinical Dementia Rating of 0 and a Mini-Mental Status Exam (MMSE) score > 25, consistent with the absence of dementia [24]. In 2013, 306 of 385 participants who had completed genotyping were included within this study. Individuals excluded from the study did not significantly differ in age, sex, race, education, or MMSE; however, they were significantly higher in their likelihood to have hypercholesterolemia than selected participants, *p* = .024. Participants were relatively healthy and cognitively intact (MMSE scores > 25), predominately female (66% female), college educated (*M* = 16.09; *SD* = 2.46), and Caucasian (96.4% and 3.6% African American). One severely obese participant (BMI = 50) was removed from analyses. Mean replacement was used for three subjects who were missing health data. 11 of the 316 subjects missing gait assessments due to technical and/or administrative errors were excluded.

The Pennington Biomedical Research Center Institutional Review Board approved all procedures included within this study. LABrainS’ procedures are conducted by well trained, certified research assistants. Participants first underwent informed consent (oral and verbal) followed by clinical interview, cognitive testing and physical measurements in a private testing suite. Subsequently, the GAITRite system [25] was administered in an adjacent well-lit hallway.

### Measures

#### Gait assessment

Gait characteristics were collected using the GAITRite system, an electronic carpeted walkway (dimensions: 90 cm X 6700 cm X 63.2 mm) with encased pressure sensors that collect information regarding the respective components that make up an individual’s walking gait. The GAITRite system provides objective, valid and reliable measurement of gait characteristics [26] and is frequently used in gait assessment of older adults in relation to cognitive changes (e.g., [3], [4], [5]). Two simple task trials were administered prior to the dual task condition. For all gait trials, participants were instructed to walk across the walkway ‘‘using their normal everyday walking speed”. Consistent with previous research, participants were instructed to spell a five-letter word backwards aloud read from a counterbalanced word list as they walked during the dual task (e.g., [4], [19], [27]). Participants were given a 2-m acceleration and deceleration distance at every trial.

#### APOE genotyping

Genomic Deoxyribonucleic acid (DNA) was extracted from blood samples by a phlebotomist at Pennington Biomedical Research Center. APOE genotyping was performed by Polymerase Chain Reaction methodology (using recommended procedures described in [28]). Participants were dichotomized into those with at least one APOE-e4 allele (*n* = 75) and individuals without an APOE-e4 allele (*n* = 231; 224 with gait data); the frequencies of homozygous APOE-e4 (*n* = 4) and APOE-e2 (*n* = 4) genotypes were rare.

#### Cognitive, health and physical assessment

The MMSE and Weschler’s Adult Intelligence Scale-Revised (WAIS-R [29]) Digit Symbol tests served as measures of cognition. The digit symbol test requires attention, processing speed, and visuomotor coordination. Notably, it has proven to be one of the most sensitive (albeit non-specific) measures for brain damage and has proven to be a good predictor of dementia progression [30]. The National Alzheimer’s Coordinating Center’s Health History clinician administered interview form collected information on presence or absence of cardiovascular disease, cerebrovascular disease, neurological (e.g., seizures and/or traumatic brain injury), biological indicators of health (hypertension, hypercholesterolemia, diabetes, thyroid disease, B12 deficiency), and psychological history. A total illness index score was calculated from the health variables. The Short Physical Performance Battery (SPPB [31]), a reliable and validated measure of lower extremity strength assessed balance, normal walking speed, and sit-to-stand time. The three timed tests were summed to create a total SPPB score (maximum score of four per test). Body mass index (BMI) was calculated from measured height (cm) and weight (kg).

### Statistical analyses

Preliminary analyses examined for group differences via ANOVAs or chi-square tests. Welch’s F-test adjusted for degrees of freedom when assumptions of homogeneity were not met.

For the primary analyses, Mixed Model ANCOVAs were used. Stride length and step time served as dependent measures during the single and dual task conditions. Sex and APOE-e4 genotype (carriers vs. non-carriers) were between subject factors, while age, height, BMI, SPPB performance, and illness index scores were covariates. All relevant statistics are reported within the paper and data are provided within S1 Dataset. Bonferroni corrections adjusted for multiple comparisons. All tests of significance were two-tailed.

## Results

### Covariates

24.7% of the participants were APOE-e4 carriers. The likelihood of men as compared to women being an APOE-e4 carrier reached a trend level difference, χ^2^ (1) = 2.86, *p* = .091. An interaction between sex and APOE-e4 indicated that female APOE-e4 carriers were significantly younger (*M* = 67.50, *SD* = 5.73) than female non-carriers (*M* = 69.87, *SD* = 6.39), *p* = .025; whereas no significant difference in age were found within male participants (*M* = 71.84, *SD* = 7.07), *p* = .332. APOE-e4 carriers as compared to non-carriers did not significantly differ in education, height, BMI, SPPB, or cognition, all *p*s > .10. Significant sex differences in age, education, height, BMI, and cognition but not SPPB performance were found (see Table 1).

**Table 1:** Sex differences in age, education, cognitive functioning, and physical assessment measures.

Individual difference variables^a^:	Men (*n* = 100)	Women (*n* = 204)	*F* =	*p* ≤
Age	71.77 (7.31)	69.25 (6.32)	9.06	.003
Years of Education	16.70 (2.48)	15.79 (2.41)	9.32	.002
Mini Mental State Exam	29.09 (1.35)	29.48 (0.97)	6.32	.013
WAIS-R Digit Symbol Coding	47.02 (9.92)	52.75 (11.14)	23.49	.001
Height in centimeters	174.41 (6.65)	160.86 (6.22)	303.46	.001
Body Mass Index	27.94 (4.04)	26.17 (4.84)	10.70	.001
SPPB^b^	11.07 (1.43)	11.09 (1.29)	.014	.907

^a^All values presented in Means and Standard Deviations: *M*(*SD*).

^b^Short Physical Performance Battery: SPPB.

Tables 2 and 3 present between group differences in health risk factors. APOE-e4 carriers as compared to non-carriers were significantly more likely to have a history of hypercholesterolemia and were overall higher on their illness index scores. Men were significantly more likely to have a history of cardiovascular disease, diabetes, and hypercholesterolemia, while females were significantly more likely to have a history of depression.

**Table 2:** Group differences in health risk characteristics by APOE-e4 carrier status.

Health Factors (%):	Total (N = 299)	APOE-e4 Carrier (*n* = 75)	Non-Carrier (*n* = 224)	*p* ≤
Cardiovascular	13.9	17.3	18.1	.887
Cerebrovascular	1.0	0	1.4	.599
Depression	27.4	32.1	25.6	.214
Diabetes	10.2	11.8	9.7	.592
Hypercholesterolemia	47.0	60.0	42.7	.009
Hypertension	48.5	55.3	46.2	.173
Total Illness Index: *M* (*SD*)	1.46 (1.24)	1.72 (1.29)	1.37 (1.51)	.039

**Table 3:** Group differences in health risk characteristics by sex.

Health Factors (%):	Men (*n* = 96)	Women (*n* = 207)	*p* ≤
Cardiovascular	27.4	10.6	.004
Cerebrovascular	2.2	.5	.281
Depression	15.6	32.9	.002
Diabetes	15.6	7.7	.035
Hypercholesterolemia	63.2	39.5	.001
Hypertension	55.2	45.4	.111
Total Illness Index: *M* (*SD*)	1.68 (1.45)	1.36 (1.12)	.056^,^^a^

^a^ Welch’s F test is reported as Levene’s statistic indicated that assumptions of homogeneity of variance were not met.

### Gait

**Fig 1:**
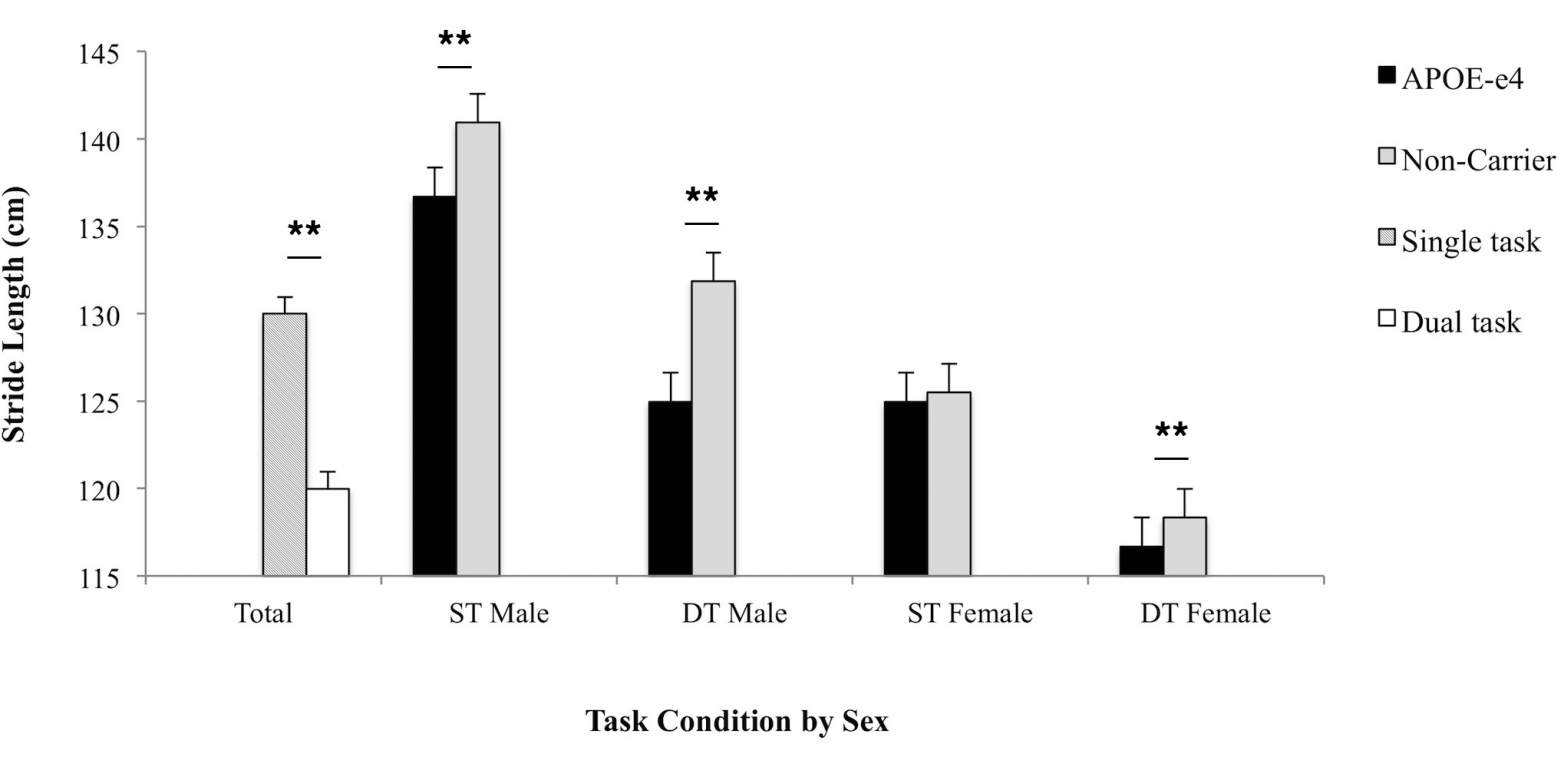
APOE-e4 carriers vs. Non-Carriers group means and standard errors for stride length (cm) by task condition and sex. Stride length was analyzed during single task (ST) and dual task (DT) conditions. Age, height, Short Physical Performance Battery, BMI and illness index scores were entered as covariates. Results represent the significant main effects of task condition and sex on stride length and the significant interaction between APOE-e4 genotype with task condition, * *p* < .05, ** *p* < .01.

Fig 1 visually presents descriptive statistics for stride length grouped by APOE-e4 carrier status and sex during both task conditions. Results indicated that stride length overall was significantly affected by age, sex, SPPB performance, height, illness index scores, and APOE-e4 genotype, such that shorter strides associated with older age, sex (women), shorter height, worse SPPB performance, greater illness, and APOE-e4 carriers across conditions, all ps < .01. No relationship between stride length and BMI was found (*p* = .876), nor was there an interaction between APOE-e4 genotype and sex, F(1,290) = .36, *p* = .545. Next, within-subject tests examined the effect of task condition on stride length with the relevant risk variables. Stride length was significantly shorter during the dual as compared to the single task condition (Mean difference = -8.66, Confidence Interval = 7.62–9.69), F(1,290) = 6.68, *p* = .010. An interaction effect between task and age suggested that older adults had a greater decrement in stride length during the dual task, F(1,290) = 7.63, *p* = .006. No significant interaction between task with sex [F(1,290) = .001, *p* = .988] or illness index scores on stride length were found [F(1,290) = .262, *p* = .604]. As hypothesized, there was a significant interaction effect between task and APOE-e4 carrier status, such that APOE-e4 carriers as compared to non-carriers demonstrated a significant greater decrement in stride length during the dual task than non-carriers, F(1,290) = 3.95, *p* = .048.

Analyses next examined the effect of task condition on step time with the relevant risk variables. Table 4 presents the descriptive statistics for step time grouped by APOE-e4 carrier status and sex during the single and dual task conditions. Results indicated that overall step time was significantly affected by age, height, and SPPB performance, all *p*s < .05. APOE-e4 genotype was not significantly associated with step time across conditions, *p* = .466. The effect of sex (females being faster) on step time across trials only reached trend, *p* = .067. BMI and illness index scores were not significantly associated with step time across conditions, all *p*s > .10. Although the simple effect demonstrated a significant difference in step time between gait task conditions (Mean difference = .022, Confidence Interval = -0.17–0.27, *p* < .001), within-subject effects for task condition only reached trend level of significance when the risk variables were adjusted for in the analyses, F(1,290) = 3.02, *p* = .083. A significant interaction between age and task condition suggested that older as compared to younger participants displayed a greater decrement in step time during the dual task, F(1,290) 5.22, *p* = .023. Similarly, an interaction between illness index scores and task condition suggested that individuals with greater illness index scores as compared to those with lower scores displayed slower step times during the dual task, F(1,290) 3.73, *p* = .044. No significant interaction between task with sex [F(1,290) = 2.03, *p* = .155] or APOE-e4 genotype on step time were found [F(1,290) = .206, *p* = .650].

**Table 4:** Group means and standard deviations for gait step time by task condition, sex and genotype.

		Men		Women		
**Step Time**^**a**^	**Total** (N = 299)	APOE-e4 (*n* = 30)	Non-Carrier (*n* = 67)	APOE-e4 (*n* = 45)	Non-Carrier (*n* = 157)	*p*^b^ =
Single Task	0.54 (.04)	0.56(.03)	0.56(.04)	0.51(.04)	0.52(.04)	.271
Dual Task	0.56 (.05)	0.59(.04)	0.58(.05)	0.54(.06)	0.55(.05)	.514

^a^Age, height, Short Physical Performance Battery, BMI and illness index scores served as covariates.

^b^P-value reflects non-significant effect of genotype on step-time.

## Discussion

This study provides the first systematic investigation of stride length and step time in relationship to demographic, genetic, and health risk factors in response to a cognitive-load walking task in non-demented older adults. As expected, differential patterns between gait characteristics with the relevant risk factors were found. Our results indicate that the effects of age, sex, and anthropometric factors need to be routinely assessed in gait studies. Stride length and step time were both influenced by older age, sex, physical performance, and height. Age also interacted with task condition indicating that advanced age is associated with greater decrements in both step time and stride length as a function of cognitive load. Greater illness burden was linked to shorter stride length across the task conditions and interacted with task to affect step time during the cognitive load task. As hypothesized, genetic risk specifically associated with stride length. APOE-e4 was a significant predictor of stride length and greater dual task decrement in stride length, but not step time.

Considering that gait is a dynamic process, these results have important implications for future research as stride length emerged as a sensitive measure of dual-task related changes in gait and was the only gait factor influenced by the presence of APOE-e4. Relevantly, reduced stride length and greater stride length variability have been linked to a higher fall risk, greater gait decrements during cognitive load dual task conditions, stroke risk and smaller hippocampal volumes with poorer memory functioning within non-demented older adult [12], [15], [16], [17], [21], [22]. Furthermore, as previously discussed, reduced stride length appears to herald changes in other gait characteristics, such as step time. In sum, our results and others suggest that stride length is a useful behavioral marker of cognitive change that is associated with genetic risk for AD.

There is research to suggest that the effects of APOE-e4 on gait decline may be specific to men and not women. Within the present study, men and women demonstrated significant differences in health related risk factors and overall gait characteristics. However, no significant genotype-sex interactions were found. Our results and others suggest that sex disparities in motor decline may be a function of medical risk factors. Such findings are relevant mechanistically considering the higher rates of cardiovascular disease, diabetes, and hypercholesterolemia found in men as compared to women, and in light of findings that link cardiovascular risk factors to decreased mobility and increased overall disability in middle-to-older adult men but not women [9]. It is possible that the APOE genotype exerts differential effects on men and women through its influence on earlier disease processes (e.g., hypercholesterolemia) that have later downstream effects. Indeed, APOE-e4 carriers were overall higher in illness index scores and significantly more likely to have a history of hypercholesterolemia. These findings and others emphasize the need for future studies that attempt to understand biological factors that may drive sex related differences in cognitive and motor declines with advanced age and whether potential indicators of early decline differ between sexes.

Potential limitations to this study include its cross-sectional design, and men and women differed in age, BMI, proportion of APOE-e4 allele carriers, education, and global cognition–which confounds the ability to make firm conclusions in regards to a sex-linked role for the APOE-e4 allele in gait decline as these factors have been shown to play a moderating role in gait changes. Additionally, LABrainS participants are generally college educated, predominantly white, with a higher proportion of women than men, which may limit the generalizability of these findings. Lastly, we cannot rule out practice effects. It is possible that certain effects may have been stronger had the conditions been counterbalanced. Overall, despite these limitations, our results are important in that they provide the first systematic investigation of gait characteristics relationship with APOE-e4 during a cognitive load dual-task.

A growing body of longitudinal research suggests that declines in motor processes are predictive of subsequent declines in cognitive functioning in older adults, and that these changes are also linked to a greater genetic risk for AD. An intriguing hypothesis that has been proposed is that sensorimotor dysfunctions may represent an endophenotype that might serve as early markers of the initiation of underlying diseases processes [32]. Although much work remains to be done, several AD risk genes have been linked to motor dysfunctions within non-demented individuals [32]. Furthermore, transgenic animal models of AD have provided causative evidence for a motor phenotype, in that overexpression of AD risk genes is associated with axonopathy within the brain and spinal cord that results in severe motor dysfunctions [33]. Consistent with this body of work, genetic risk for AD was linked to shorter stride lengths even when other relevant variables were assessed. All considered, assessment of gait characteristics in relation to genetic risk for AD might increase the sensitivity by which older adults at risk for a dementia disorder are detected. However, while this idea holds promise, there has been limited research on the underlying neural mechanisms involved in gait disturbances. Future research should continue to delineate the relationship between changes in neural substrates and specific gait characteristics in older adults and determine whether risk profiles differ between men and women, as these may inform future pharmacological and behavioral interventions for AD, as well as other dementia disorders.

## Supporting Information

S1 Dataset (CSV)
